# Pre-mRNA processing includes *N*^6^ methylation of adenosine residues that are retained in mRNA exons and the fallacy of “RNA epigenetics”

**DOI:** 10.1261/rna.065219.117

**Published:** 2018-03

**Authors:** Robert B. Darnell, Shengdong Ke, James E. Darnell

**Affiliations:** 1 Laboratory of Molecular Neuro-Oncology, The Rockefeller University, New York, New York 10065, USA; 2 Laboratory of Molecular Cell Biology, The Rockefeller University, New York, New York 10065, USA

## Abstract

By using a cell fraction technique that separates chromatin-associated nascent RNA, newly completed nucleoplasmic mRNA and cytoplasmic mRNA, we have shown in a previous study that residues in exons are methylated (m^6^A) in nascent pre-mRNA and remain methylated in the same exonic residues in nucleoplasmic and cytoplasmic mRNA. Thus, there is no evidence of a substantial degree of demethylation in mRNA exons that would correspond to so-called “epigenetic” demethylation. The turnover rate of mRNA molecules is faster, depending on m^6^A content in HeLa cell mRNA, suggesting that specification of mRNA stability may be the major role of m^6^A exon modification. In mouse embryonic stem cells (mESCs) lacking Mettl3, the major mRNA methylase, the cells continue to grow, making the same mRNAs with unchanged splicing profiles in the absence (>90%) of m^6^A in mRNA, suggesting no common obligatory role of m^6^A in splicing. All these data argue strongly against a commonly used “reversible dynamic methylation/demethylation” of mRNA, calling into question the concept of “RNA epigenetics” that parallels the well-established role of dynamic DNA epigenetics.

## INTRODUCTION

Producing eukaryotic mRNA, especially in animal cells, requires many biochemical steps including 5′ capping, 3′ polyadenylation, obligatory and alternative splicing of many exons and base modifications, the latter of which has been much discussed recently with respect to *N*^6^ adenosine methylation of residues that exist in mRNA (He 2010; Fu et al. 2014; Wang and He 2014; Cao et al. 2016; Ke et al. 2017; Meyer and Jaffrey 2017; Roundtree et al. 2017). To reflect more directly on the recent mRNA methylation studies and commentaries, a look back at earlier and recent techniques and conclusions is in order.^3^

A central question was originally, and has remained, when in the formation of an mRNA do any required biochemical modifications of pre-RNA occur (for review, see Darnell 2013)? Only in 1994 did a cell fractionation technique emerge that liberated for study unfinished, nascent pre-mRNA *still* attached to polymerase and chromatin. Wuarin and Schibler (1994) prepared liver cell nuclei and released this chromatin fraction by dissolving the nuclei in a solution containing 1 M urea. Using specific hybridization of labeled DNA probes, they established that in two specific pre-mRNAs some but not all of the introns in the still attached nascent RNA were removed. This experiment was the first indisputable evidence that splicing can and does occur in pre-mRNA before transcription is complete in mammalian cells and provides the opportunity to determine when in the course of mRNA formation m^6^A methylation occurs.

### *N*^6^ adenosine methylation in mRNA

m^6^A residues in mRNA were discovered in 1974 in cultured mouse cells by Robert Perry, a pioneer in early mammalian mRNA studies, and his colleague Dawn Kelley (Perry and Kelley 1974). The quantitative measurement of an average of one m^6^A residue per ∼1000 nucleotides (nt) in mRNA (Perry and Kelley 1974), and the presence of m^6^A in cellular mRNA was confirmed by others (Desrosiers et al. 1975; Furuichi et al. 1975a,b; Wei et al. 1975). Also, m^6^A was found in large nuclear poly(A)^+^ mRNA (now known to be pre-mRNA) (Salditt-Georgieff et al. 1976). m^6^A was also found in adenovirus infected cells in both viral mRNA (Sommer et al. 1976, 1978) and pre-mRNA (Chen-Kiang et al. 1979). Bokar et al. (1994) discovered the likely methylase and cloned the first mRNA methylase (Bokar et al. 1997). James Manley and colleagues established the favored oligonucleotide context for m^6^A addition (Harper et al. 1990). This much about m^6^A in mRNA was known by 1997. However, little was learned about the details of when in the formation of cellular mRNA the *N*^6^ methylation actually occurred or what the function of m^6^A in RNA might be.

Based on new technology, the methylation of *N*^6^ on adenosine residues in pre-mRNA and mRNA has attracted much recent attention. Highly specific m^6^A antibodies (Munns and Sims 1975) only became commercially available after 2010, allowing precipitation of m^6^A-containing mRNA fragments followed by sequencing of the precipitated fragments. The initial sequencing experiments showed wide distribution of m^6^A residues in mRNA (Dominissini et al. 2012; Meyer et al. 2012) with a heavy concentration in the last exon (Ke et al. 2015) not close to (within ∼50 nt of) a STOP codon but mostly in the 3′ UTR (untranslated region). These latter experiments examined RNA that was UV cross-linked to anti-m^6^A antibody followed by RNA sequencing (HITS-CLIP) (Licatalosi et al. 2008), and m^6^A-induced truncation site analysis has allowed precise localization of m^6^A modified residues (Ke et al. 2017).

### Chromatin liberation and nascent RNA isolation coupled with HITS-CLIP analysis for m^6^A residues

The Wuarin–Schibler technique for isolation of the chromatin fraction and the attached nascent RNA was adapted to cultured cells by Douglas Black and his graduate student, Amy Pandya-Jones, in 2009 (Pandya-Jones and Black 2009). Extensive studies from the Black (Pandya-Jones et al. 2013) and Steve Smale (Bhatt et al. 2012) laboratories then traced the formation of seven toxin-induced mRNAs in macrophages from nascent RNA to a nucleoplasmic fraction and then to the cytoplasm, establishing an accurate time course and the extent of processing in each fraction. In every case, processing was complete before the release into the nucleoplasm with both poly(A) addition and splicing completed in the chromatin fraction. Poly(A) was apparently added in most cases before the last splicing was completed and all nucleoplasmic molecules were completely processed.

Together with Jones and Black, we investigated the time of m^6^A appearance in HeLa cell mRNA formation (Ke et al. 2017). It was very clear that m^6^A methylation occurred on the nascent RNA, in many instances *before* splicing had occurred. Adenosine residues in exons received at least 98% of the methylation despite at least a threefold excess of intron to exon residues being present in the nascent RNA. Furthermore, the nascent RNA introns had many more informatically predicted target sites for m^6^A than the exons. The distribution of m^6^A residues within the exons in the pre-mRNA strongly favored the last exon and the majority of these were within the 3′ UTR, as was observed earlier in the total cytoplasmic mRNA (Ke et al. 2015). As emphasized below, m^6^A location in pre-mRNA exons included all sites in the fully processed nucleoplasmic and cytoplasmic mRNA, arguing strongly against obvious and frequent separate m^6^A editing activity after completion and release of the nascent pre-mRNA.

### Important role of m^6^A in mRNA turnover but not splicing

The major functional role we identified for a great majority of the m^6^A residues in mRNA was in increasing the turnover time, *T*_1/2_, of m^6^A bearing molecules (Ke et al. 2017). There was a general coincidence of increasing turnover of an mRNA with increasing m^6^A content. While no single m^6^A residue was shown to be required for mRNA turnover, the correlation of fast turnover time with m^6^A content suggests most m^6^A residues in mRNA function in mRNA instability.

We also examined mouse embryonic stem cells (ESC) missing the major methylase Mettl3 (by genetic knockouts generated by Shay Geula and Jacob Hanna [Geula et al. 2015; Ke et al. 2017]). The Mettl3^–^ ESC cells generated by Geula and Hanna continue to grow indefinitely in culture, and produce virtually *all* the mRNAs the wild-type parental cells produce, as Geula et al. (2015) had described earlier. We found that mRNA splicing in the Mettl3^−^ cells was essentially indistinguishable from normal cells even though they had only 10% of normal m^6^A methylation. This result contradicts the claim of Dominissini et al. (2012). That group described a pervasive effect on splicing of a Mettl3 knockdown of HepG2 cells. However, the analysis was on day 6 of the knockdown when cells were described as severely damaged (Dominissini et al. 2012). Moreover, using their published data, we could not confirm the pervasive splicing changes described. While it remains possible that some splicing events might require m^6^A, such an occurrence remains to be demonstrated. It seems safe to state that the vast majority of m^6^A residues *do not* participate in splicing and in HeLa cells they are not concentrated near splice junctions (Ke et al. 2017). Translation of the majority of mRNAs in the Mettl3^–^ ESCs must be reasonably normal since the mutants lacking 90% of m^6^A grow normally (Geula et al. 2015; Ke et al. 2017). However, it must be kept in mind that the ESC have lost regulation of changes necessary for differentiation. Since the mRNAs for transcription factors are among those heavily methylated (Ke et al. 2017), it would be reasonable to expect a critical balance of transcription factors to be upset (Geula et al. 2015).

### Specific function of m^6^A residues at 5′ end of mRNAs

Recently, a few specifically targeted effects of m^6^A addition to residues in the 5′-end region of mRNAs have been described. These are the *only* single m^6^A residues to have a specific function demonstrated at the moment. Heat shock is known to cause the formation of new mRNAs (Lindquist and Craig 1988; de Nadal et al. 2011) and several of these newly synthesized heat shock mRNA molecules were found to have m^6^A residues in the untranslated residues near the 5′ end (Meyer et al. 2015; Mitchell and Parker 2015; Zhou et al. 2015). The new m^6^A methylated mRNAs are translated at elevated temperature likely because of the demonstrated attraction of the eukaryotic initiation factor 3 (eif 3) for the methylated residues. It is NOT known where in the cell this m^6^A is added in these mRNAs, but it is known that some heat shock mRNAs are newly transcriptionally induced and could acquire the m^6^A as nascent pre-mRNA.

Recently reported is a second m^6^A modification exactly at the 5′ end of an mRNA that controls stability. Decades ago it was found that the first nucleotide Pol II incorporated in a pre-mRNA can be adenylic acid (∼40% of the time). This residue is, of course, acted upon by several enzymes to create a 5′ cap and is also frequently methylated in the *N*^6^ position (Furuichi et al. 1975a,b; Wei et al. 1975). Mauer et al. (2017) have now found that this *N*^6^ methylated adenosine (m^6^Am) cap residue can be stable on specific mRNAs. The demethylase FTO (and not ALKBH5) will remove this m^6^Am residue in vitro. Further, in these in vitro experiments the FTO was more active on the m^6^A in the cap than on the m^6^A in the body of the mRNA. Also, this enzyme is required to carry out this demethylation in the cell, upon which the mRNA is destabilized and turns over using the decapping pathway of turnover (Wang et al. 2002). Of interest, the FTO demethylase was much less effective on the m^6^A residues in the body of the mRNA in the cell than the ALKBH5 enzyme in vitro. The knockout of the ALKBH5 enzyme in mice shows a requirement for spermiogenesis and has no other reported effect, strong testimony that no obligatory methylation/demethylation is widely used in most cells (Zheng et al. 2013a).

The FTO action on m^6^Am is the first known specific demethylase removal of a specific adenosine methylation and at present the only direct evidence of a function for modification of a single m^6^A residue. The removal of the cap m^6^Am is assumed to be cytoplasmic but the addition of the methyl groups is likely nuclear. Also, the 5′ heat shock-stimulated m^6^A addition discussed above may well occur during the formation of a new RNA molecule in the nucleus.

### Role and cellular location of other demethylations

What is known about any larger role of demethylation of m^6^A in pre-mRNA or mRNA? The recognition that the FTO gene product contains an amino acid sequence similar to active domains in DNA demethylases spurred the hypothesis and subsequent demonstration by the C. He group that the protein is indeed an RNA *N*^6^ adenosine demethylase (Jia et al. 2011). This group then made the in silico recognition of ALKBH5 as a likely second RNA m^6^A demethylase which proved to be true (Fu et al. 2010; Zheng et al. 2013a,b). The presence of both RNA methylases (so-called “writers”) and demethylases (so-called “erasers”) then led to the oft-repeated, as yet unfounded, speculation that the m^6^A methylation–demethylation represents a “dynamic epigenetic” regulatory system that underlies an important previously unrecognized regulatory circuit for the general control of information flow from DNA to mRNA (see the title of Zheng et al. 2013b and Fu et al. 2014).

This speculation of RNA epigenetics was first announced in 2010 (He 2010), modeled after the *known* and proven “epigenetic” changes in histone modification that take part in governing pre-mRNA transcription. The idea continues to be presented in the title of a 2014 review/speculation/proposal: “Gene expression regulation mediated through reversible m^6^A RNA methylation” (Fu et al. 2014). To quote from the 2010 paper: “Given the rich layers of epigenetic regulation that result from targeted modifications of DNA and proteins, *reversible RNA modification might* (emphasis added) represent another realm for biological regulation in the form of ‘RNA epigenetics’.” (He 2010). This same point of view continues in Fu et al. (2014) (see Abstract, the final sentence of which is “This reversible RNA methylation adds a new dimension to the developing picture of post-transcriptional regulation of gene expression.”)

This idea has been so frequently repeated in published papers, largely by the C. He group and by “science writers” in leading journals (Willyard 2017), that it has caused this speculation to seem in some quarters to be established as fact (Fu et al. 2014; Wang and He 2014; Cao et al. 2016; Dominissini et al. 2016; Gilbert et al. 2016; Roundtree et al. 2017).

C. He and colleagues have published a number of substantial findings on mRNA methylation: Of particular note is recognition of RNA demethylases (Jia et al. 2011; Zheng et al. 2013a,b); proof that the demethylases act only on single-stranded nucleic acids (Jia et al. 2011); the binding of the YTHDF2 protein to m^6^A methylated mRNA thought to be connected to mRNA turnover (Wang et al. 2014); and in cataloging and proving several other base modifications on mRNA (Roundtree et al. 2017). But upon critical examination of what is now known about m^6^A residues in mammalian pre-mRNA and RNA, the central notion of an “RNA epigenetics” paralleling DNA methylation and demethylation and especially histone methylation/demethylation and phosphorylation/desphosphorylation and the effects on gene expression is only a speculation and hence as put forth, seriously misleading to uncritical readers. The reversibility (forward or backward) attached to a specific event/capacity is missing (see Allis et al. 2008 for DNA methylation and also refer to Meyer and Jaffrey 2017).

Our data show that in HeLa cells the m^6^A sites in nucleoplasmic and cytoplasmic mRNA are already methylated in nascent chromatin-associated specific pre-mRNA transcripts (Ke et al. 2017). If there were active cytoplasmic m^6^A mRNA methylases or demethylases, they would necessarily be constrained to target the same sites as in nascent pre-mRNA. The m^6^A residues in steady-state cytoplasmic mRNAs show no specific new sites or detectable demethylation of specific m^6^A residues that were added to the nascent RNA in the nucleus.

Our conclusion is that the methylation of m^6^A in nascent pre-mRNAs on particular residues remains on these residues until the mRNA turns over in the cytoplasm and is NOT subject to “epigenetic” changes. Fu et al. (2014) lament the lack of evidence of cytoplasmic demethylation in their 2014 review; however, they conclude: “Although both FTO and ALKBH5 are mainly found in the nucleus, the possibility that both proteins could translocate to the cytoplasm under certain circumstances should not be ruled out. Cytoplasmic RNA may also be demethylated by these enzymes or by other currently unknown demethylases.”

Several papers dealing with demethylases have shown (mainly with fluorescent antibodies) that these proteins may be largely nuclear (Jia et al. 2011; Zheng et al. 2013a). However, the authors are at great pains to direct the reader's attention to the fact that specific demethylase antibodies show a high concentration of these proteins in “nuclear speckles.” They infer activity on nuclear RNA of the demethylases *because* of this location. But it has long been established that “speckles” contain many molecules, e.g., factors known to act in splicing, that are not active in splicing while in the speckles (Spector and Lamond 2011). Splicing occurs on nascent RNA on chromatin (for review, see Darnell 2013). In contrast, speckles are storehouses for processing proteins (recognized earlier as intra-chromatin granules). The pre-mRNA processing almost certainly occurs on what are referred to by electron microscopists as perichromatin filaments NOT in the nuclear speckles (see Spector and Lamond 2011).

Did we learn anything enlightening about nuclear demethylation? Perhaps. We found in HeLa cells ∼3600 out of ∼30,000 m^6^A sites scattered throughout pre-mRNA exons that did not survive when these molecules are processed, released, and reach the nucleoplasm. Further, these “disappearing” m^6^A sites were in ∼10% of specific pre-mRNAs (Ke et al. 2017). It is possible that this m^6^A loss could be due to the previously mentioned m^6^A demethylases (Jia et al. 2011; Zheng et al. 2013a). Even if it is true that specific pre-mRNA nascent molecules undergo partial demethylation in the nucleus before completion of processing, this nuclear demethylation is hardly evidence of a “dynamic” methylation–demethylation in the cell at large *especially on mRNA*. There is no credible evidence of a meaningful “on and off” m^6^A reversibility let alone any proof of a regulatory event. Rather, such likely nuclear demethylation as described above is possible on specific nascent transcripts in HeLa cells and represents a necessary removal of a methylation for the correct nuclear processing and/or function of that particular mRNA when it reaches the cytoplasm. The possible function of this small set of nuclear methylations and apparent demethylations surely requires additional investigation. But the present results are hardly the foundation for a regulatory “RNA epigenetics” that is “dynamic.”

### Time for biochemistry of mRNA turnover mediated by the internal m^6^A residues in mRNA

Biochemists have known for years that different mRNAs in eukaryotic cells exhibit different turnover times (Parker and Song 2004; Garneau et al. 2007; Song et al. 2010; Schoenberg and Maquat 2012; Wu and Brewer 2012). The main function for *most* of the m^6^A residues in mRNA in mammalian cells appears to be increasing mRNA turnover since a higher m^6^A content in an mRNA molecule correlates with faster turnover (Ke et al. 2017). The m^6^A residues are concentrated in about half of mRNAs (56% have two or more); 17% have only a single m^6^A, and 27% have *no* m^6^A residues. Perhaps clusters or m^6^A in near neighbors in an mRNA play a role in turnover. Finally, it is important to reiterate that approximately a quarter of mRNAs in HeLa cells have no m^6^A residues but these molecules also have a range of half-lives similar to the range in m^6^A-containing mRNAs (Ke et al. 2017). Considerable progress has been made in recent times on the enzymes involved in mRNA turnover (Parker and Song 2004; Garneau et al. 2007). It seems likely that one or more of these pathways may rely on m^6^A to promote turnover of some methylated mRNAs.

Wang et al. (2014) demonstrated that methylated mRNAs can be bound by YTHDF2 proteins, to which they assign a role in turnover because this protein presumably with its mRNA cargo in tow is found in cytoplasmic P bodies, which are known to contain proteins (decapping and deadenylation enzymes) active in mRNA turnover. There is an obvious need to learn what role the m^6^A plays in cooperation with the mRNA turnover machinery and why a higher content of m^6^A plays a role in the turnover rate of specific mRNA. Some highly methylated mRNAs show clusters but this clustering is not always present (Ke et al. 2017).

There are a few specifically identified functions of m^6^A residues in mRNAs that reside in the 5′ end. m^6^A methylations are required in the 5′ UTR for translation of newly induced heat shock mRNAs (Meyer et al. 2015; Zhou et al. 2015). Second, some mRNAs contain m^6^A on the initiating and capped adenosine residue stabilizing that mRNA. Removal by the FTO methylase leads to turnover of such mRNAs (Mauer et al. 2017). These two findings indicate that some m^6^A residues can have a role in translation and in protecting mRNAs to be translated. No more general role in translation has been demonstrated but could yet be discovered.

### CONCLUSION

m^6^A residues are scattered throughout mammalian mRNA but concentrated in the last exon and 3′ UTR. These methylations are definitely added to exons in the nucleus. After pre-mRNA conversion to mRNA, the great majority of these methylations remain in place in the cytoplasm and somehow contribute directly to the turnover of mRNA in the cytoplasm. The biochemical basis for this participation in cytoplasmic mRNA turnover is unknown.

While m^6^A demethylases exist and appear to erase specific m^6^A groups on a limited set of nascent pre-mRNAs, no clear function at the cytoplasmic mRNA level has yet been assigned to this demethylation. Two quite different single m^6^A methylations at or near the 5′ end of mRNAs affect mRNA translation in one case and mRNA stability in the other. There is at the present time no evidence for functional addition/removal, i.e., a reversible m^6^A regulatory pathway, in the majority of mRNA molecules of mammalian cells.

## Footnotes

3 A very thorough and illuminating review of the m^6^A field was just published online (Meyer and Jaffrey 2017) that concurs with our major conclusion that the “RNA epigenetics” proposal is at best premature. Quote: “The idea that m^6^A in mRNA is reversible has been an important concept. Conceivably, the activation of m^6^A demethylases could account for dynamic changes in m^6^A levels in mRNA. However, despite excitement around the concept, very few studies have explored this concept at the molecular level.”
